# Fine Structure of Antennal Sensilla of *Paysandisia archon* and Electrophysiological Responses to Volatile Compounds Associated with Host Palms

**DOI:** 10.1371/journal.pone.0124607

**Published:** 2015-04-23

**Authors:** Sara Ruschioni, Paola Riolo, Elisa Verdolini, Ezio Peri, Salvatore Guarino, Stefano Colazza, Roberto Romani, Nunzio Isidoro

**Affiliations:** 1 Dipartimento di Scienze Agrarie, Alimentari ed Ambientali, Università Politecnica delle Marche, 60131 Ancona, Italy; 2 Dipartimento di Scienze Agrarie e Forestali, Università degli Studi di Palermo, 90128 Palermo, Italy; 3 Dipartimento di Scienze Agrarie, Alimentari ed Ambientali, Università degli Studi di Perugia, 06121 Perugia, Italy; 4 Istituto per la Protezione Sostenibile delle Piante-CNR, 50019 Sesto Fiorentino (FI), Italy; INRA-UPMC, FRANCE

## Abstract

*Paysandisia archon* (Lepidoptera: Castniidae) is a serious pest of palm trees. A comprehensive knowledge of the insect olfactory system is essential for the development of efficient semiochemical-based control methods. The olfactory sensilla are located particularly on the antennae, and these can detect plant volatiles that provide important cues for the insects in the search for their host plants. To date, the fine structure of *P*. *archon* antennal sensilla studies and their role in host-plant perception have not been investigated in great detail. Using light microscopy and scanning and transmission electron microscopy, the antennae of both sexes of *P*. *archon* are described here in detail, according to the different types, quantities and distributions of the sensilla. Six types of sensilla were identified. The most widespread are sensilla trichoidea, sensilla basiconica and sensilla auricilica, which are associated with olfactory function. These have cuticular shafts characterised by numerous pores, and they are innervated by two or three sensory neurons. Sensilla coeloconica, sensilla chaetica and sensilla ampullacea are associated with olfactory or olfactory-thermoreception, mechano-gustatory, and thermo-hygroreception functions, respectively. Moreover, the role of *P*. *archon* antennae in locating of the host palms was evaluated using electroantennograms, to monitor responses to ester and terpene compounds previously identified as volatiles of damaged/fermenting palm tissues. *P*. *archon* showed responses to all of the synthetic chemicals tested, with greater responses in the females, providing a significant sex*dose effect. Among the compounds tested, ethyl isobutyrate elicited the strongest antenna responses. The fine structure of the cuticular and cellular components of the *P*. *archon* antenna sensory equipment is described for the first time. The results of this study form an important starting point and complement physiological and behavioural studies, to provide valuable information of practical importance for the development of efficient semiochemical-based control methods.

## Introduction

*Paysandisia archon* Burmeister (the palm borer moth [PBM]) (Lepidoptera: Castniidae) is a phytophagous species that is highly specialised to feed only on palms (Aracaceae family) [1]. Although the PBM has not been reported to be a significant pest in its native area (South America) [1, 2], in Europe it is causing serious damage and plant mortality in both palm nurseries and urban areas [3]. It is currently on the European and Mediterranean Plant Protection Organisation (EPPO) A2 List (N° 338) of “Pests recommended for regulation as quarantine pests” [3].

The PBM is extremely difficult to control because, except for a very short time after egg hatching, its larvae are endophagous, and therefore chemical control has only limited efficacy [4]. The larvae penetrate deep into the stem and damage its internal tissues, disrupt nutrient transport, and even lead to tree collapse and death [1]. Integrated pest-management strategies are moving more towards semiochemicals, which are informative molecules used in insect-insect or plant-insect interactions. This can thus be considered as an alternative or complementary approach to insecticide treatments [5].

To date, no castniid female sex pheromone is known, although the female of another castniid species, *Telchin* (syn *Castnia*) *licus* (Drury), has also been investigated for the identification of pheromone compounds. Indeed, hexane extracts of *T*. *licus* ovipositors have been analyzed by gas chromatography-mass spectrometry and they were shown to elicit male responses in bioassays [6]. PBM adults are day-flying insects that fly in hot sunny weather, and they are inactive under cloudy or rainy weather conditions [1]. Recent studies have described a perching mate-locating behaviour of PBM males, with the female triggering the courtship sequence by approaching the perching male first [7], with visual cues involved in the courtship behaviour, and a lack of any female long-range sex pheromone [7, 8]. Moreover, two different putative short-range male pheromones were identified in extracts from the male wings [as a mixture of Z,E-farnesal, E,E-farnesal, and (E,Z)-2,13-octadecadienol] [8], and the male mid-legs [(E,Z)-2,13-octadecadienol] [9], and the intensity of the electroantennogram (EAG) responses with PBM male and female antennae was about 0.2 mV [8, 9]. Sarto i Monteys et al. (2012) used scanning electron microscopy (SEM) images to describe four types of PBM antennal sensilla (chaetica, trichoidea, basiconica, auricilica), and they reported that the morphology of these PBM antennae are strikingly similar to those of butterflies [8]. Indeed, in butterflies (i.e., Rhopalocera), the antennae usually consist of a long flagellum with a distal bulb that forms a club that comprises the last 10–12 enlarged antennomeres. Moreover, no sexual dimorphism in antennal structure has been reported in diurnal Lepidoptera [10–12].

Insect antennae are peripheral sensory structures that are involved in the detection of many important environmental stimuli [13]. They carry a wide range of sensilla types that serve a multiciplicity of sensory modalities, including olfaction, gustation, mechanoreception, thermoreception and hygroreception [13–15]. The antenna olfactory sensilla house receptor neurons that can recognize odours from foods and plants, and can detect pheromones that are released by conspecifics [16]. Reception of these volatile compounds is mediated by olfactory receptor neurons, which act to convert the chemical signals into electrical signals that input directly into the central nervous system of the insect [16]. The combined electrophysiological responses of thousands of differentially and narrowly tuned olfactory receptor neurons on the insect antennae provide the whole antenna responses to odours, which are known as EAGs [17, 18]. These EAGs can reveal sensitivities to a wide variety of odorants, including host-plant odour volatiles that are important in behaviour patterns [19–22].

Although some aspects of the reproductive biology (e.g., sexual maturity, diel periodicity of mating, occurrence of polyandry and delay between mating and laying eggs.), courtship and mating behaviour of PBM are known [7–9, 23], there remains a lack of information as to the mechanisms that drive their host-plant selection. Host-plant odours can have an important role in the host selection processes of the female insects, which will thus affect the survival and distribution of their offspring, especially when the immature stages have little opportunity to change their developmental location [24]. Preliminary observations (P. Riolo, personal observations) have revealed that PBM females prefer to oviposit on palms damaged by PBM larvae rather than on undamaged palms. Plants respond to herbivore feeding through increased biosynthesis and emission of volatile compounds from the damaged tissues; i.e., terpenes and esters [25]. Terpenes can act as a host-plant attractants [26–28], or repellents [29], or as pheromones or synergists to a pheromone [30]. Similarly, esters can act as host-plant attractants [31–33], or repellents [33], or as synergists to a pheromone [34, 35].

The aim of the present study was to provide the first detailed fine-structure characterisation of the cuticular and cellular components of the PBM antenna sensory equipment, using light microscopy, SEM and transmission electron microscopy (TEM). Furthermore, the numbers of antennomeres were counted, and the different typologies, quantities and distributions of the sensilla were investigated. The lengths of the sensilla trichoidea were also measured, to determine any differences between the sexes. To assess the responsiveness of the antennae to host volatiles, dose-response bioassays were carried out, using: (i) linalool (terpene), a major compound of the palm volatiles that has been identified in flowers and leaves [36, 37]; and (ii) a range of esters, including ethyl acetate, ethyl propionate, ethyl butyrate, ethyl isobutyrate and ethyl lactate, that have been identified as the main compounds of damaged and fermenting palm-tissue volatiles [35]. These data provide an important starting point and are complementary to further physiological and behavioural studies on the PBM and other Lepidoptera species. Moreover, comprehensive knowledge of the PBM olfactory system is essential for the development of efficient semiochemical-based control methods.

## Materials and Methods

### Insects

Adult PBMs were obtained from potted 2-3-year-old infested plants of *Chamaerops humilis* (L.) that were purchased from nurseries in the municipality of Grottammare (42°59’22”N; 13°51’56”E), in the Province of Ascoli Piceno in the Marche Region (central Italy). The infested palms were placed in a net tunnel inside a greenhouse in the municipality of Ancona (43°35’11”N; 13°30’50”E) in the Marche Region, at the Dipartimento di Scienze Agrarie, Alimentari ed Ambientali, Università Politecnica delle Marche (Italy). The pupae were stored in a climate chamber (KBWF 240; Binder, Tuttlingen, Germany), at 15 ±1°C, under 24 h darkness and 60% relative humidity. A stock of pupae was periodically removed from this storage and kept at 25 ±1°C, under 16 h light, 8 h dark and 60% relative humidity, to promote adult emergence.

### Morphology and fine structure investigations

#### Light microscopy

Light microscopy was used to count the number of antennomeres. The antennae were excised from the heads of newly emerged adults, cold anaesthetized. The scales were removed and observed under a stereo microscope (MZ 125; Leica, Wetzlar, Germany) (n = 20; sex ratio, 1:1).

#### Scanning electron microscopy (SEM)

**Fig 1:**
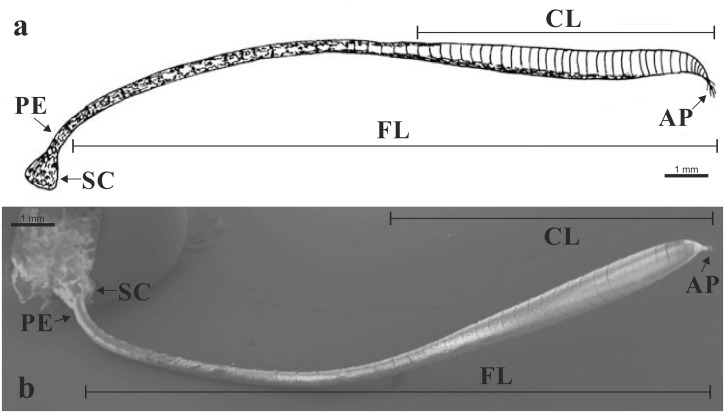
Antenna of *Paysandisia archon*. (a) Schematic drawing; (b) SEM overall view of a male antenna. [SC] scape; [PE] pedicel; [FL] flagellum; [CL] club; [AP] apiculus. Scale bars: 1 mm (a).

The SEM images were used to determine the morphology of the antennae, including for the different typologies, quantities and distributions of the sensilla, and for measurement of the lengths of the sensilla trichoidea. The antennae were removed from the head capsule of newly emerged adults, cold anaesthetised (n = 40; sex ratio, 1:1). The specimens were dehydrated through a series of graded ethanol, from 50% to 99% (Sigma Aldrich; Milan, Italy), and mounted on aluminum stubs, taking care to place them with different orientations, to obtain views of the ventral and dorsal aspects, and of both of the lateral sides. The mounted specimens were gold sputtered (Union SCD 040, Balzers, FL, USA), and examined under SEM (Supra 40; Zeiss; Oberkochen, Germany). Forty sensilla trichoidea, were randomly chosen from five adults of each sex, and measured for their length, using Smart SEM software (V05.04: 08-may-09; Zeiss, Oberkochen, Germany) (n = 400; sex ratio, 1:1). To study the distribution of the sensilla, each antennal club (Fig 1) was divided into four sections of equal length from the tip to the base of the club (n = 20; sex ratio, 1:1), as: C1, distal section; C2–C3, intermediate sections; C4, proximal section. In each section counting of sensilla was carried out over an area of 2500 μm^2^ (50 μm × 50 μm) (here defined as the ‘unit area’).

#### Transmission electron microscopy (TEM)

The antennae were isolated and immediately immersed in a solution of 1% glutaraldehyde and 2.5% paraformaldehyde in 0.1 M cacodylate buffer with 5% sucrose (pH 7.2–7.3) [38]. The specimens were cut into small fragments (2–3 flagellomeres) to aid fixative penetration, and left at 4°C for 2 h. After rinsing overnight in 0.1 M cacodylate buffer, the fragments were post-fixed in 1% OsO_4_ for 1 h at 4°C, and then rinsed in the same buffer. Dehydration of these antenna fragments was through a graded ethanol series, which was followed by embedding in Epon-Araldite, with propylene oxide as the bridging solvent. Thin sections (100 nm) were cut with a diamond knife (Diatome; Biel/Bienne, Switzerland) on an ultramicrotome (Leica Ultracut R; Wetzlar, Germany) and mounted on formvar-coated 50-mesh grids. Finally, after staining with uranyl acetate (15 min, room temperature) and lead citrate (5 min, room temperature), the sections were examined under electron miscroscopy (Philips EM 208; Eindhoven, The Netherlands). All of the chemicals were purchased from Sigma Aldrich (Milan, Italy). Digital pictures were taken using a high-resolution digital camera (MegaView III; Soft Imaging System GmbH; Münster, Germany) connected to the electron microscope. Ten newly emerged cold-anaesthetised adult PBMs of each sex were used for these ultrastructural investigations.

#### Electroantennograms (EAGs)

Dose-response recordings of the antenna responses of both sexes of the PBM were conducted using the synthetic chemicals ethyl acetate, ethyl propionate, ethyl butyrate, ethyl isobutyrate, ethyl lactate and linalool (all purities >95%), all of which were purchased from Sigma Aldrich (Milan, Italy). These compounds were serially diluted 1:10 in hexane (purity 99%), down to a concentration of 1 μg μL^-1^. Then, 1 μL aliquots of each tested compound were used for each active ingredient, using the amounts of 1, 10, 100 and 1000 μg. The antennae were excised from the heads and were suspended above two silver-wire electrodes using glass capillary tubes filled with 0.1 M KCl solution. A standard 1 μL aliquot of each test solution was pipetted onto a piece of filter paper (Whatman, grade 1), exposed to the air for 20 s to allow the solvent to evaporate, and then inserted into a glass Pasteur pipette. A stimulus flow controller (model CS-05; Synthech; Hilversum, The Netherlands) was used to generate a 1.5-s stimulus at 1-min intervals, with a flow rate of 1.5 L min^-1^ [39]. The signals generated by the antennae were passed through a high-impedance amplifier (model IDAC-232; Synthech; Hilversum, The Netherlands) and recorded with specialised software (EAG Pro, Synthech; Hilversum, The Netherlands). The same antenna was used to test all of the concentrations of a single compound. Each compound was tested on 12 antennae (sex ratio, 1:1) using one antenna per moth. The sequence of the tested compounds was randomised, and was provided starting from the weakest concentration and followed by increasing concentrations. At the beginning and end of the stimulation of each antenna with each concentration of the six compounds, 1 μL pure hexane was puffed, as reference.

The EAG response elicited from hexane (i.e., the mean of the two puffs at the beginning and at the end of each set of stimulus doses) was subtracted from the responses obtained by the test stimuli in order to normalise the responses. The approach taken was based on preliminary experiments where the EAG responses to hexane elicited in PBM males (0.04 ± 0.01 mV; n = 6) and females (0.06 ± 0.01 mV; n = 6) were compared, with no significant differences observed (F = 0.95; P = 0.35; df = 1).

### Data analysis

To calculate the total number of each sensillum type on an entire antenna, we divided the sensillar area (2,860,000 μm^2^) by the unit area, and multiplied by the number of each sensilla type in the unit area (n = 20; sex ratio, 1:1). Sex differences for club length, diameter and area, and the number of antennomeres were analysed using one-way ANOVA. Differences in sensilla lengths and number/unit area between sexes and club sections were analysed using one-way ANOVA followed by Tukey’s tests for mean separation; all of these data were log-transformed to meet the assumption of normality.

The EAG responses to the different doses of chemicals for the male and female antennae were analysed by repeated measures ANOVA, with sex and dose as independent variables; mean EAG responses were separated using least significant difference tests.

All of the statistical tests were performed using the Systat 11 software (Systat Software Inc., San Jose, CA; SPSS 2000).

### Ethics statement

This study investigated a moth that is not an endangered or protected invertebrate species. All of the necessary permits were obtained for the studies described.

## Results

### Antennal morphology and fine structure

The PBMs show segmented clubbed antennae that comprise the scape, pedicel and flagellum. The scape is the first basal antennal segment, and it is articulated with the head through the torulus, and connected by an elastic joint membrane. The pedicel (second segment) is articulated proximal to the scape and distal to the rest of the antenna (flagellum). The flagellum is composed of several antennomeres that gradually enlarge (from the 12^th^ antennomere) towards the distal part, forming a club (from about the 18^th^ antennomere). The distal part of the flagellum is an apiculus that consists of the last 5–10 segments, and appears tapered and to curve slightly upwards: it has numerous long setae at the tip (Fig 1). The number of antennomeres is not significantly different between the sexes (F = 0.93, P >0.05, df = 1; Table 1).

**Table 1:** Number of antennomeres and club measurements for the *Paysandisia archon* antennae.

Measure	Males	Females
Antennomeres (n)	58.70 ±1.11	57.40 ±0.78
Club length (mm)	5.52 ±0.16	6.58 ±0.17***
Club maximum diameter (μm)	658.2 ±4.7**	618.1 ±12.3
Club total area (mm^2^)	11.41 ±0.32	12.79 ±0.46*
Club basal diameter (μm)	330.7 ±7.4	336.1 ±7.3

Data are means ±SE (n = 20; sex ratio 1:1)

*P <0.05

**P <0.01

***P <0.001, significant differences for male *versus* female comparisons (one-way *ANOVA* test).

Some 80% of the antenna surface is covered with enlarged and distally dentate scales; the rest of the surface is without scales and has instead numerous sensilla, therefore defining the sensillar area. This sensillar area starts as a non-continuous ventral strip at the level of the 12^th^ to 17^th^ antennomere, and then it becomes more uniform for about 10 antennomeres, and progressively enlarges towards the tip. About 85% of the total number of sensilla is located on the ventral side of the club.

The club length is significantly greater in females than in males (F = 19.93, P <0.001, df = 1; Table 1), while the opposite is observed for the maximum diameter of the club (F = 9.34, P <0.01, df = 1; Table 1). The total club area is significantly greater in females than in males (F = 6.09, P <0.05, df = 1; Table 1). There is no significant difference in the basal diameters of the clubs between the sexes (F = 0.28, P >0.05, df = 1; Table 1).

The SEM and TEM investigations of these PBM antennae revealed the following six types of sensilla: trichoidea, basiconica, auricilica, coeloconica, chaetica and ampullacea. Sensilla trichoidea, sensilla basiconica and sensilla auricilica are the most numerically abundant over the entire sensillar area, while there are lower numbers of sensilla coeloconica, sensilla chaetica and sensilla ampullacea.

From the tip to the base of the club, the number/unit area of sensilla trichoidea in males and females decrease significantly (males: F = 30.44, P <0.001, df = 3; females: F = 17.50, P <0.001, df = 3; Table 2), as also for sensilla basiconica in females (F = 12.28, P <0.001, df = 3; Table 2). In males, in the centre of the club, there is significantly greater number/unit area of sensilla basiconica (F = 4.81, P <0.01, df = 3) and sensilla auricilica (F = 5.91, P <0.01, df = 3), while in females, the sensilla auricilica number/unit area is uniform throughout the club (F = 1.61, P >0.05, df = 3; Table 2). The sensilla trichoidea/sensilla basiconica ratio and the sensilla trichoidea/ sensilla auricilica ratio are 1.56 and 2.56, respectively, in females, and 4.07 and 6.76, respectively, in males.

**Table 2:** Sensilla trichoidea, sensilla basiconica and sensilla auricilica in the different sections of the *Paysandisia archon* antenna club.

Antenna club section^§^	Sensilla (sensilla n/unit area)					
	Trichoidea		Basiconica		Auricillica	
	Males	Females	Males	Females	Males	Females
C1	12.60 ±0.52a	11.60 ±0.67a	2.20 ±0.25a	7.90 ±0.57a	1.20 ±0.13a	3.30 ±0.26a
C2	10.50 ±0.31b	8.10 ±0.60b	3.30 ±0.26b	4.30 ±0.47b	1.00 ±0.15a	3.80 ±0.49a
C3	9.40 ±0.31b	6.80 ±0.65bc	2.10 ±0.23a	3.70 ±0.30b	2.40 ±0.34b	3.00 ±0.58a
C4	7.40 ±0.40c	5.70 ±0.52c	2.20 ±0.29a	4.80 ±0.71b	1.30 ±0.33a	2.50 ±0.31a
**Overall means**	9.98 ±0.36**	8.05 ±0.46	2.45 ±0.15	5.18 ±0.36***	1.48 ±0.15	3.15 ±0.22***

Data are means ±SE (n = 20; sex ratio 1:1)

*P <0.05

**P <0.01

***P <0.001, significant differences in male *versus* female comparisons (one-way *ANOVA* test)

Different letters within the same column indicate statistical differences (Tukey’s test, P <0.05)

^**§**^ C1, distal section; C2–C3, intermediate sections; C4, prossimal section

**Fig 2:**
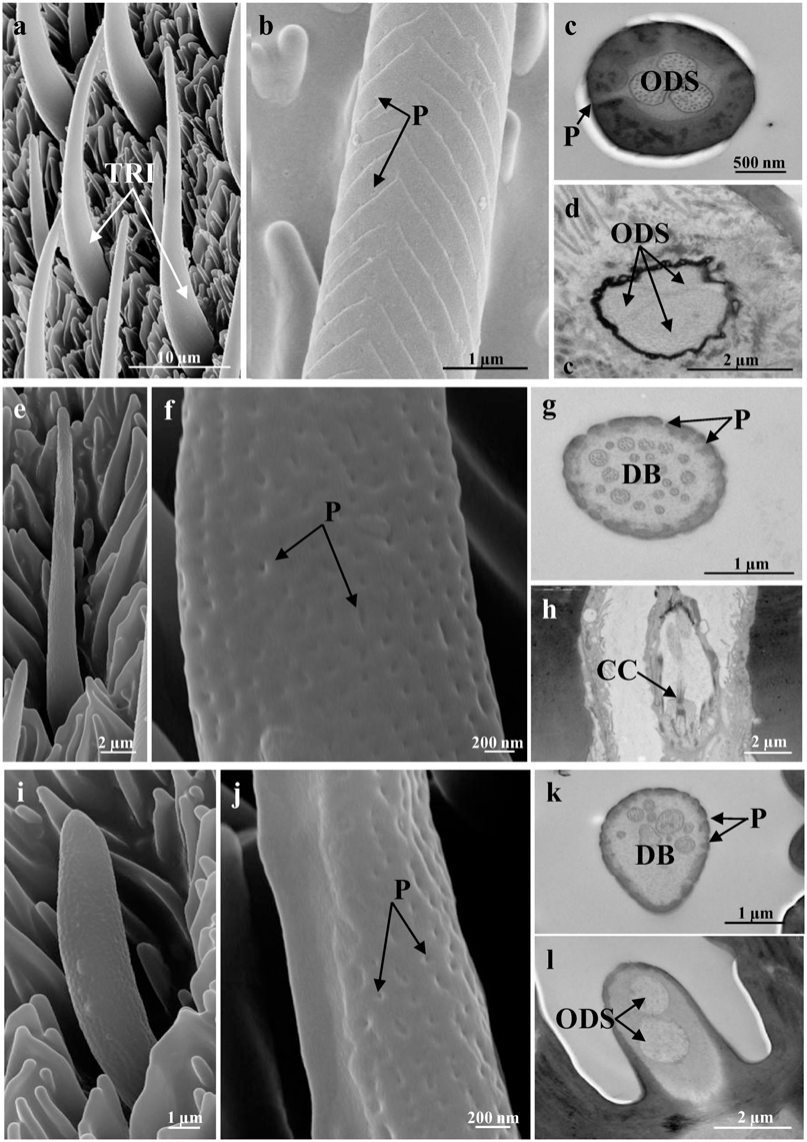
Representative SEM (a, b, e, f, i, j) and TEM (c, d, g, h, k, l) images of the more numerous sensilla. (a-d) Sensilla trichoidea, showing low-magnification details (a), herringbone grooves and pores [P] (b), and cross-sections of the shaft with thick-walled cuticle [P] and outer dendritic segments [ODS] with three sensory neurons (c), and of the base with three sensory neurons enclosed in a common dendritic sheath [ODS] (d). (e-h) Sensilla basiconica, showing low-magnification details (e), the numerous pores [P] (f), cross-section of the shaft with the thin-walled cuticle [P] and the dendritic branches [DB] (g), and an oblique section of the base with the ciliary constriction [CC] (h). (i-l) Sensilla auricilica, showing low-magnification details (i), the numerous pores [P] (j), cross-section of the shaft with the thin-walled cuticle [P] and dendritic branches [DB] (k), and an oblique section of the base, with two sensory neurons enclosed in a common dendritic sheath [ODS] (l). Scale bars: 10 μm (a); 2 μm (d, e, h, l); 1 μm (b, g, i, k); 500 nm (c); 200 nm (f, j).

Sensilla trichoidea are characterised by an elongated cuticular shaft that decrease in diameter towards the apex (Fig 2a). There are numerous pores dorsally along the herringbone grooves (Fig 2b and 2c). The cuticular shaft is inserted into the antennal wall through an inflexible socket. The TEM images show a thick-walled sensillum that is innervated by two or three sensory neurons (Fig 2c and 2d), with three accessory cells. The outer dendritic segments of the sensory neurons are enclosed in a common dendrite sheath (Fig 2d); however after entering the peg lumen, they remained unbranched to the tip of the shaft (Fig 2c). Among the main typologies (sensilla trichoidea, sensilla basiconica, sensilla coeloconica), the most abundant are sensilla trichoidea, with significantly higher proportions both in males (71% of the total sensilla; F = 379.48, P <0.001, df = 2) and females (49% of the total sensilla; F = 46.15, P <0.001, df = 2). The number/unit area of sensilla trichoidea is also significantly higher in males than in females (F = 10.93; P <0.01; df = 1; Table 2). Despite this, as the sensillar area is larger in females, the total number of sensilla trichoidea is lower in males. The sensilla trichoidea lengths are not significantly different between the sexes (males: 44.99 ± 4.22 μm; females: 44.41 ± 4.69 μm; F = 0.10, P >0.05, df = 1).

Sensilla basiconica are characterised by a thin elongated cuticular shaft, which is about 20 μm long, and in which there are numerous pores (Fig 2e–2g). These pores are distributed over the entire surface without any apparent specific distribution pattern. At their base, sensilla basiconica do not have a flexible socket, and they are surrounded by the antenna wall. The TEM images show the thin cuticular wall that is pierced by numerous minute pores, and the dendritic branches of two to three sensory neurons (Fig 2h). Proximally, the outer dendritic segments of the sensory neurons are enclosed in a common dendrite sheath (Fig 2g). Sensilla basiconica represent 18% and 32% of the total number of the main sensilla typologies in males and females, respectively. Their number/unit area is significantly higher in females than in males (F = 47.93, P <0.001; df = 1; Table 2).

Sensilla auricilica are characterised by an elongated and laterally flattened cuticular shaft that is about 12 μm long (Fig 2i). The cuticular wall is covered by numerous pores that are evenly distributed (Fig 2i–2k). Also in this case, the sensilla auricilica are inflexible. The TEM images show the thin-walled sensillum that is innervated by two sensory neurons (Fig 2l). The sensory neurons enter the peg lumen as outer dendritic segments that are enclosed in a common dendritic sheath, and they branch when they reach the tip of the shaft (Fig 2k). Sensilla auricilica represent 11% and 19% of the total number of the main sensilla typologies in males and females, respectively; their number/unit area is significantly higher in females than males (F = 39.40, P <0.001; df = 1; Table 2).

**Fig 3:**
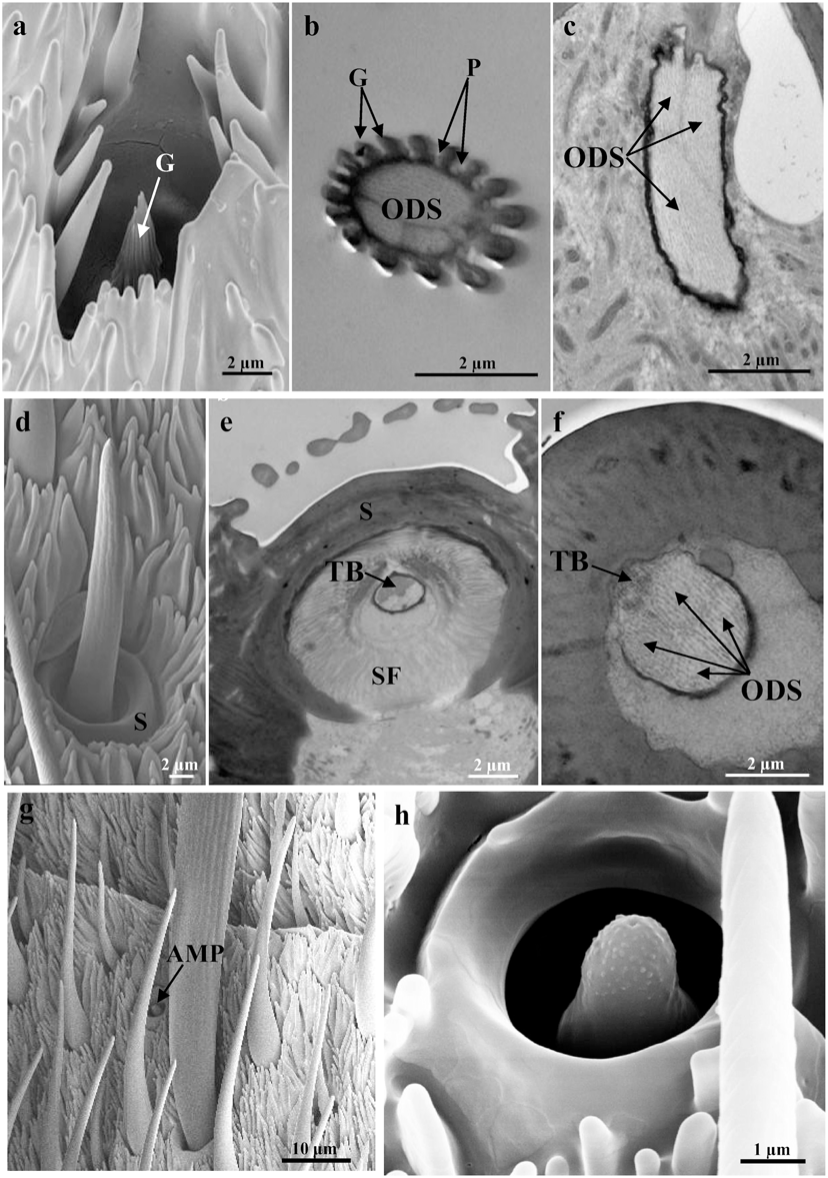
Representative SEM (a, d, g, h) and TEM (b, c, e, f) images of the less numerous sensilla. (a-c) Sensilla coeloconica, showing low-magnification details (a), and cross-section of the shaft with grooves [G], pores [P] and the outer dendritic segments [ODS] (b), and oblique section of the base with three outer dendritic segments [ODS] enclosed in a common dendritic sheath (c). (d-f) Sensilla chaetica, showing low-magnification details (d), and cross-section of the socket [S] with the joint membrane, suspension fibres [SF], and tubular body [TB], (e) and of the base with the tubular body [TB] and four outer dendritic segments [ODS] (f). (g, h) SEM images showing the sensilla ampullacea. Scale bars: 10 μm (g); 2 μm (a-f); 1 μm (h).

Sensilla coeloconica have a cuticular part that is a small and clavate peg. This peg is inserted into the antennal wall without a flexible socket, and proximally it has a smooth cuticle. Distally, about 16 finger-like cuticular projections have developed, giving a grooved appearance to the peg (Fig 3a and 3b). There are many pores along the grooves, except closer to the tip (Fig 3b). The peg, which is about 5 μm long, is completely embedded within the antennal wall, and it sit in an ellipsoid-shaped cavity that is about 6.5 μm wide (Fig 3a). This cavity is shallow and wider at the bottom than at its opening (Fig 3a). The TEM images show the typical double-walled coeloconica sensillum that has three sensory neurons associated with it (Fig 3b and 3c). The peg lumen is completely occupied by the unbranched outer dendritic segments of the sensory neurons, which reach to the tip of the sensilla coeloconica (Fig 3b and 3c). There are no differences in the number of sensilla coeloconica between males and females. In some antennomeres, there are up to eight sensilla coeloconica located mainly in the lateral sensillar area near the scales, and their distribution does not appear to follow any fixed pattern, as they are not uniform across the specimens.

Sensilla chaetica are characterised by an elongated cuticular shaft that is inserted into the antennal wall through a flexible socket (Fig 3d and 3e). This socket shows a joint membrane with the development of suspension fibres (Fig 3e). The hair shaft diameter decreases towards its rounded tip (Fig 3d). At this level, there is a single apical pore. These sensilla are about 20 μm in length, and they have a thick cuticular wall. The TEM images show that the cellular components consist of five sensory neurons (Fig 3f). Four of these sensory neurons enter the peg lumen as outer dendritic segments enclosed in a common dendrite sheath, and these reach the tip of the shaft without branching. The outer dendritic segment of the fifth sensory neuron ends at the base of the sensilla chaetica, in a tubular body that is attached to the joint membrane (Fig 3e and 3f). Sensilla chaetica are located at the base of the antennomeres, generally on all of the antennomeres, with variable numbers that range from one (at the level of the last five antennomeres), up to five on the other antennomeres.

Sensilla ampullacea appear as dome-shaped pegs that are about 3 μm long, and are completely embedded within the cuticular cavities (diameter, about 3.5 μm), without any pores in the cuticular wall (Fig 3g and 3h). As seen for sensilla coeloconica, the sensilla ampullacea are located in the lateral sensillar area near the scales. Their distribution does not appear to follow any fixed pattern; only up to two sensilla ampullacea are found and on a limited number of antennomeres.

### Electroantennograms

**Fig 4:**
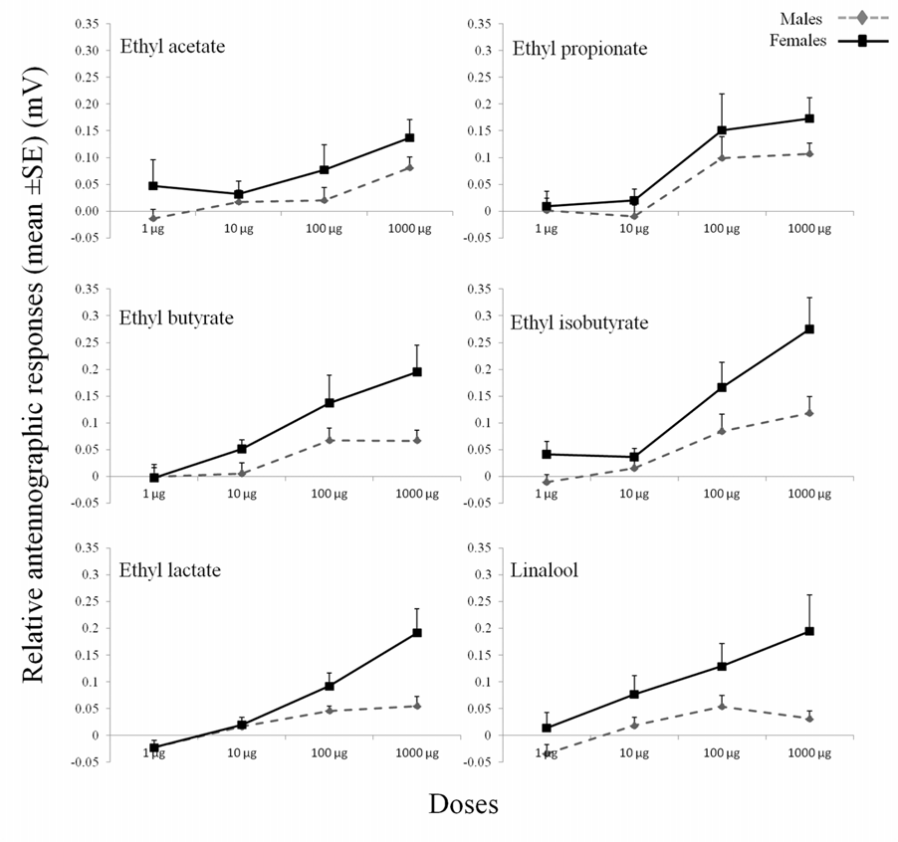
EAG dose—response curves for the antennae of *Paysandisia archon* males and females in response to the synthetic chemicals. EAG amplitudes were adjusted to the control stimulus (hexane), and are given as means ±SE. Each chemical was tested on six individuals of each sex.

The antennae of the PBM adults showed responses to all of the synthetic chemicals tested (Fig 4). Significant differences in the EAG responses were observed between the sexes (F = 29.56, P <0.001, df = 1) and among the doses (F = 52.65, P <0.001, df = 3). In particular, a significant sex*dose effect (F = 6.19, P <0.001, df = 3) was observed, with the female responses higher than the male responses with the increasing of the doses: the sex differences in the EAG responses were more evident at the dose of 1000 μg (Fig 4). The highest EAG response was recorded with ethyl isobutyrate at the 1000 μg dose for both the males and the females. The dose of 1000 μg of ethyl butyrate, ethyl isobutuyrate, ethyl lactate and linalool showed significantly higher responses in females (P <0.05). No significant sex*chemical (F = 0.52, P >0.05, df = 5,) or dose*chemical (F = 1.14, P >0.05, df = 15) effects were seen.

## Discussion

PBMs have segmented clubbed antennae that comprise the scape, pedicel and flagellum. According to Sarto i Monteys *et al*. (2012), these antennae are thin and club shaped without any evident sexual dimorphism. Their sensillar area is extremely reduced, and it is mainly on the ventral side of the club [8], as observed usually for day-flying butterflies [40, 41]. The antennomeres of both the males and the females are cylindrical in shape, and their diameters enlarge gradually from the 12^th^ antennomere, and quickly decrease for the very last segments before the apiculum. The ventral surface of the club has numerous sensilla, with numerous overlapping scales covering the rest of the antennae. This arrangement of distinct ‘sensory’ and ‘scale’ surfaces has been reported for several lepidopteran species belonging to different families: *Danaus gilippus berenice* (Cramer) [10], *Euphydryas editha* (Boisduval) [11], *Aglais urticae* (L.), *Polygonia c-album* (L.) [12] (Nymphalidae), *Synanthedon tipuliformis* (Clerck) [42] (Sesiidae), *Spodoptera exigua* (Hübner) [43], *Helicoverpa assulta* (Guenée) [44] (Noctuidae), *Chilo partellus* (Swinhoe) [45], *Zamagiria dixolophella* Dyar [46] (Pyralidae), and *Talponia batesi* (Heinrich) [47] (Tortricidae). The assumed function of this arrangement includes protection of the antennae and their sensilla from damage [44], contributions to the ability to detect the direction of a stimulus [48], and/or a mechanism to trap and concentrate odorant molecules [49, 50].

The present study describes for the first time the fine structure of the cuticular and cellular sensilla components of the PBM antennae. This is supported by both SEM and TEM analyses, which revealed the presence of six types of sensilla: trichoidea, basiconica, auricilica, chaetica, coeloconica and ampullacea. This study has shown that sensilla trichoidea are the most abundant and widespread over the entire sensillar area. They are the only sensillum type with a number/unit area that is higher in the male; however, despite this higher number/unit area, as the sensory surface is larger in the female, the total number of sensilla trichoidea is lower in the male. Some Lepidoptera (e.g., Pyralids, Sesids, Noctuids) have been showed to have different types of sensilla trichoidea, with different lengths for the same types of sensilla trichoidea between the sexes [45, 51, 52]. The PBM sensilla trichoidea resemble the “long thin-walled pegs” described in *D*. *gilippus berenice* and *Colias* spp. (Pieridae) [10, 53], and the “sensilla type II (shallow dish sensillum)” in *E*. *editha* [11]. Our observations show that with the PBM, the lengths of this single type of trichoidea sensilla do not vary between the sexes. Several studies have associated these sensilla trichoidea with the detection of olfactory stimuli [15, 54, 55], and Hillier *et al*. (2006) demonstrated that in *Heliothis virescens* F. (Noctuidae) female there are plant-odour-detecting neurons in the sensilla trichoidea [56].

We observed that for the PBM antennae, the number/unit area of the sensilla basiconica is significantly greater in the females than the males. The electron microscopy images shown in this study are similar to the studies carried out on *D*. *gilippus berenice* (where they reported short, thin-walled pegs) [10], *H*. *assulta* [44], and *Scoliopteryx libatrix* (L.) [57] (Noctuidae), and these numerous pores suggest that the sensilla basiconica of the PBM function as olfactory chemoreceptors. In the literature, sensilla basiconica have been shown to be involved in both host odour detection and pheromone detection [58–60]. In the Arctiid moth, *Utetheisa ornatrix* (L.), the sensilla basiconica in the females are unexpectedly sensitive to the male-produced pheromone, as the receptor neurons responsive to insect pheromones in male moths appear, as a rule, to be associated with the pored wall of the sensilla trichoidea [61, 62].

The PBM sensilla auricilica are innervated by two sensory neurons and they have thin multiporous walls, as observed also in *S*. *libatrix* [57] and *Cydia pomonella* (L.) [63] (Tortricidae). There is a greater number/unit area of sensilla auricilica in the PBM females, compared to the males. Studies on Noctuidae [57] and Tortricidae [63, 64] species have shown that sensilla auricilica are involved in plant odour detection, and in *C*. *pomonella* with the detection of minor components of the sex pheromone [65]. In the literature, there is no evidence for sensilla auricilica on Rhopalocera species.

In PBM, sensilla coeloconica appear as short double-walled sensilla set in a pit, and with three sensory neurons. These sensilla could be referred to as the ‘multiporous grooved pegs’ that have been reported for many insect Orders [15, 66, 67]. These were found scattered in low numbers near to the surface covered by scales. Sensilla coeloconica have often been reported as being grouped in small patches in specific antennal areas [67–69]. In *D*. *gilippus berenice*, sensilla coeleconica were found grouped in spherical subcuticular chambers, with a number ranging from 25 to 80 pegs [10]. However, in the Nymphalid *E*. *editha* the “sensillum type III (hidden sensillum)” showed a structure very similar to the PBM sensillum coeloconicum [11]. These sensilla coeloconica have usually been associated with olfactory function or with a double olfactory-thermoreception function [13, 14, 70]. In different lepidopteran families, sensilla coeloconica are housing neurons sensitive to plant odours [14], like in *C*. *partellus* [45], *Bombyx mori* (L.) [68] (Bombycidae), and *Plutella xylostella* (L.) [71] (Plutellidae).

Several studies have suggested that sensilla chaetica have both contact chemoreception and mechanoreception functions, as they arise from a socket and have a terminal pore [14, 48]. The sensilla chaetica structure that we describe here, with the tubular body and the four outer dendritic segments that enter the peg lumen, suggests that their role involves both mechanoreception and contact chemoreception [13]. As for butterflies, sensilla that are morphologically and functionally analogous to the sensilla chetica have been described and referred to as “long thick-walled hairs”, in *D*. *gilippus berenice* [10], *Colias eurytheme* Bisduval and *C*. *philodice* Godart [53], while in *E*. *editha*, the term “sensilla type I (the spine) was used [11].

The present study thus shows that sensilla ampullacea in PBMs are distributed on both lateral sides of the antennomeres. They are embedded within the antenna wall, and are closely related to the no-pore sensilla [72] that have been described for many insect Orders [13].

Plants release a large number of volatile compounds, and one approach to select candidate compounds for behavioural tests is to screen these volatiles for their antennal activity. This is often carried out by EAG recordings [19–22]. Lepidopteran females primarily need to use these olfactory cues to determine the suitability of oviposition sites and the presence of potential competitors or co-habitants [73]. In our study, female PBM antennae showed greater sensitivity to the compounds tested that are potentially involved in host-moth interactions compared to the male antennae, in agreement with what has been observed in *Manduca sexta* (L.) [74] (Sphingidae), *Cnaphalocrocis medinalis* (Guenée) and *Marasmia patnalis* Bradley [75] (Pyralidae), *Heliconius melpomene* L. [76] (Nymphalidae), *Pieris rapae* (L.) [21] (Pieridae), and *Graphium sarpedon* (L.) [77] (Papilionidae). This differential odour sensitivity could be attributed to the presence of specific receptors that respond to specific categories of chemicals, as in the case of the female *M*. *sexta* antennae [78]. Moreover, the EAG bioassays carried out in the present study showed dose—response effects for all of the compounds tested. EAG response amplitudes to general host plant volatiles similar to those recorded for the PBM here have been recorded in *C*. *pomonella* [79, 80], *M*. *sexta* [74] and *H*. *melpomene* [76].

Among the Lepidoptera species, attraction to fermenting tissue volatile compounds has been reported for different noctuid and torticid species [32]. The effects of intraspecific insect infestation on host plants have been studied also in *Mamestra brassicae* (L.) [81] (Noctuidae) and in *Ectropis obliqua* (Prout) [82] (Geometridae), which showed that mated females are more attracted to conspecific damaged plants. However, further studies need to be carried out to determine whether the EAG-active compounds are indeed involved in host plant-PBM interactions, and also whether they have any role in the field.

The present study has an important role, as it has provided the first fundamental information on the fine structure of the cuticular and cellular components of the PBM antenna sensory equipment. Although the PBM belongs to a moth family, its antennae show striking similarities to those of day-flying butterflies (i.e., no sexual dimorphism, clubbed structure, reduced sensillar area where the different sensilla are concentrated). This is of great relevance in terms of the evolution of different communication strategies between the two main Lepidoptera groups, e.g. butterflies (diurnal, based mainly on visual cues) and moths (nocturnal, based mainly on pheromone production/detection).
